# Interfering with mitochondrial dynamics sensitizes glioblastoma multiforme to temozolomide chemotherapy

**DOI:** 10.1111/jcmm.17147

**Published:** 2021-12-28

**Authors:** Nan Wang, Renxuan Huang, Kunmeng Yang, Yichun He, Yufei Gao, Delu Dong

**Affiliations:** ^1^ China‐Japan Union Hospital Jilin University Changchun China; ^2^ The First Hospital of Jilin University Changchun China; ^3^ The Basic Medical College of Jilin University Changchun China

**Keywords:** AMPK, glioblastoma multiforme, mitochondrial dynamics, temozolomide, TP53

## Abstract

Glioblastoma multiforme (GBM) is a primary tumour of the central nervous system (CNS) that exhibits the highest degree of malignancy. Radiotherapy and chemotherapy are essential to prolong the survival time of patients. However, clinical work has demonstrated that sensitivity of GBM to chemotherapy decreases with time. The phenomenon of multi‐drug resistance (MDR) reminds us that there may exist some fundamental mechanisms in the process of chemo‐resistance. We tried to explore the mechanism of GBM chemo‐resistance from the perspective of energy metabolism. First, we found that the oxidative phosphorylation (OXPHOS) level of SHG44 and U87 cells increased under TMZ treatment. In further studies, it was found that the expression of PINK1 and mitophagy flux downstream was downregulated in GBM cells, which were secondary to the upregulation of TP53 in tumour cells under TMZ treatment. At the same time, we examined the mitochondrial morphology in tumour cells and found that the size of mitochondria in tumour cells increased under the treatment of TMZ, which originated from the regulation of AMPK on the subcellular localization of Drp1 under the condition of unbalanced energy supply and demand in tumour cells. The accumulation of mitochondrial mass and the optimization of mitochondrial quality accounted for the increased oxidative phosphorylation, and interruption of the mitochondrial fusion process downregulated the efficiency of oxidative phosphorylation and sensitized GBM cells to TMZ, which was also confirmed in the *in vivo* experiment. What is more, interfering with this process is an innovative strategy to overcome the chemo‐resistance of GBM cells.

## 1 INTRODUCTION

Glioblastoma multiforme (GBM) is the most malignant primary tumour of the nervous system (CNS), with a very poor prognosis. The progression‐free survival of patients with this condition after diagnosis is 6.2–7.5 months; the median survival is 14.6–16.7 months^1^, ^2^, ^3^ and the 5‐year survival rate is only 10%.^4^ The current standard therapy for GBM is the maximal surgical resection with postoperative radiotherapy and chemotherapy.^5^ However, the postoperative recurrence of GBM is inevitable and the survival period of patients with recurrence is usually no more than 12 months.^6^, ^7^ Currently, the first‐line chemotherapy agent used for GBM is temozolomide (TMZ), an imidazole tetrazine alkylating agent.^8^ However, clinical observations have shown that the sensitivity of GBM to TMZ treatment decreases over time and that this may be closely related to multiple inherent or acquired mechanisms that confer resistance to chemotherapy.

In the studies of the chemo‐resistance, mitochondria are under spotlight for a long time. Mitochondria are involved in vital processes such as energy synthesis, regulation of calcium ion, ROS generation and apoptosis execution^9^, which all play roles in chemo‐resistance. In addition, the mitochondrial quality control system also affects the sensitivity of tumour cells to chemotherapy.^10^

As we all know, TMZ kills tumour cells by inducing DNA damage, and the survival cells are always with upregulated DNA damage response or multiple stress responses. Although these responses vary, energy support is essential.^8^ That's why we took the energy supply in glioma cells under TMZ treatment as subject in this study. Under conditions of chemotherapeutic stress, the metabolic pattern of tumour cells undergoes reprogramming in order to adapt to cellular stress status.^11^ In this study, we found that the metabolic reprogramming of tumour cells involved in mitochondrial dynamics is a mechanism underlying the resistance of GBM to TMZ. Mitochondrial dynamics is an important promoter of metabolic reprogramming. Mitochondrial dynamics consists of a range of processes, including mitochondrial fission, fusion and mitophagy.^12^ Mitochondrial fission excludes severely damaged mitochondria from the mitochondrial system, while mitochondrial fusion can achieve structural and functional complementation between damaged mitochondria,^13^ thereby affecting cell metabolism by controlling the quantity and quality of the mitochondria. Interfering with the process of mitochondrial dynamics and the metabolism in tumour cells can sensitize GBM to TMZ. The study provides us with new ideas for the treatment of GBM.

## 2 MATERIALS AND METHODS

### 2.1 Reagents and antibodies

TMZ, Compound C, AICAR, Mdivi1, MG132 and WY14643, were purchased from MedChemExpress company. MTT and N‐Acetyl‐L‐cysteine (NAC) were purchased from Sigma‐Aldrich. MitoTracker™ Red FM (M22425), Hoechst 33342 (H1399) and anti‐Ubiquitin WB Antibody (13–1600) were purchased from Invitrogen (Thermo Fisher Scientific, Inc.) (1:1,000). Anti‐phospho‐Ubiquitin (Ser65) (ABS1513‐I) was purchased from Merck KGaA company (1:1000). Anti‐P53 (21891–1‐AP), anti‐PINK1 (23274–1‐AP), anti‐Parkin (14060–1‐AP), anti‐Drp1 (12957–1‐AP), anti‐Opa1 (27733–1‐AP), anti‐Mfn1 (13798–1‐AP), anti‐Mfn2 (12186–1‐AP), anti‐Caspase‐9 (10380–1‐AP), anti‐BAX (50599–2‐Ig), anti‐Caspase‐3 (19677–1‐AP), anti‐VDAC1 (10866–1‐AP), anti‐Lamin B (12987–1‐AP), anti‐Cytochrome c (10993–1‐AP), and anti‐β‐actin (60008–1‐Ig) were purchased from ProteinTech Group, Inc., (Chicago, IL, USA) (1:1,000). Anti‐γ‐H2A.X (ab81299) was purchased from Abcam (1:1000). Anti‐AMPKα (2532) and p‐AMPKα (50081) were purchased from Cell Signaling Technology, Inc. (Massachusetts, USA) (1:1000). Anti‐phospho‐DRP1(Ser616) (DF2972) was purchased from Affinity Biosciences (1:1000). Anti‐phospho‐DRP1(Ser637) (6319S) was purchased from Cell Signaling Technology, Inc. (1:1,000).

### 2.2 Cellular and animal models

Human glioblastoma cell lines (SHG44, U87MG and U251MG) were obtained from the Chinese Academy of Medical Sciences and cultured in Dulbecco's modified Eagle's medium supplemented with 10% foetal calf serum, penicillin (50 U/ml) and streptomycin (50 µg/ml) purchased from Life Technologies and incubated at 37°C in a 5% CO_2_ atmosphere.

### 2.3 Animal models

The experimental protocol was approved by the local ethics committee (20200921–1). Nude mice were adapted to the experimental environment for a week prior to experimentation. Then, 5 × 10^6^ U87MG cells were suspended in 100 µl of phosphate buffer saline (PBS) (Beyotime Institute of Biotechnology) and injected subcutaneously into the right armpit of the nude mice. The animals were raised in a specific pathogen‐free (SPF) environment, and tumour volume was observed regularly. One week later, when the tumour volume reached 50 mm^3^, the nude mice were randomly divided into four groups, with eight mice in each group. The treatment conditions for each group were as follows: control group: 200 µl of 5% Carboxymethyl Cellulose (CMC)—daily gavage; TMZ group: 40 mg of TMZ suspended in 200 µl of 5% CMC—daily gavage; WY14643 group: 10 mg of WY14643 suspended in 200 µl of 5% CMC—daily gavage; TMZ+WY14641 group: TMZ (40 mg) + WY14643 (10 mg) suspended in 200 µl of 5% CMC—daily gavage. The status of the nude mice was observed daily. Mice were weighed and the size of each tumour was measured every 2 days. The experiment was terminated when the longest diameter of the tumour reached 2 cm or signs of ulceration appeared. Upon termination of the experiment, the nude mice were sacrificed, and the tumour was stripped, photographed and weighed. The tumour tissue was used for the following experimental tests: (1) evaluating the ATP content of tissue cells; (2) the total protein was extracted from the tumour tissue for Western blotting (WB); (3) part of the tumour tissue was stored in 2.5% dialdehyde and analysed by transmission electron microscopy; and (4) part of the tumour tissue was stored in 4% paraformaldehyde for immunohistochemical analysis.

### 2.4 Western blot protein analysis

Cells were lysed in Prusiner's buffer (Tris‐HCl 1 M; pH 7.5 containing NaCl (150 mM), EDTA (5 mM), Triton ×‐100 (0.5%), and deoxycholate and protease inhibitor cocktail). The homogenates obtained were then briefly sonicated. Aliquots of 10µg of total protein were then loaded onto 8%–16% SDS‐PAGE gels. After migration, proteins were wet‐transferred to PVDF membranes and immunoblotted using certain antibodies. Immunological complexes were detected with either anti‐rabbit or anti‐mouse IgG‐coupled peroxidase antibodies (ProteinTech Group, Inc.) by the electrochemiluminescence detection method (Roche Diagnostics S.A.S).

### 2.5 Immunofluorescence assay

Cells were grown on coverslips in 24‐well plates and treated with different agents. After staining with MitoTracker for 30 min, the coverslips were rinsed with PBS and fixed with 4% (w/v) paraformaldehyde for 25 min at room temperature followed by a PBS rinse. Permeabilization with 0.1% (v/v) Triton ×‐100 for 7 min was followed by blockade with 10% horse serum for another 30 min. Following incubation with primary antibodies (1:100) at 4°C for 12 h, the coverslips were incubated with fluorescent secondary antibodies (1:200) for 30 min, rinsed with cold PBS and stained with Hoechst 33342 in accordance with the manufacturer's instructions for 7min before a final PBS rinse. A Revolve Hybrid Microscope (Discover ECHO) was used for image capture. Images were further processed by ImageJ Software version 1.52 s (National Institutes of Health) to assess the intensity of immunofluorescence (IF), as well as the mitochondrial parameters. For the evaluation of mitochondrial fusion, we evaluated three images in each treatment group from three independent experiments using ImageJ Software. Two parameters were used to assess the level of mitochondrial fusion: the mean size of the mitochondrial network (MS) and the mean length of mitochondria (ML).

### 2.6 Measurement of glucose and lactate concentration

Cells were seeded in 6‐well plates at a density of 3 × 10^5^ cells/well. Cells were then incubated overnight at 37°C, and the medium was replaced with fresh complete medium. After 24 h, the culture medium was collected and proteins were extracted by sonication. Extracted proteins were then quantified with a Bradford Protein Assay kit (Beyotime Institute of Biotechnology). Then, we measured the concentrations of glucose and lactate with glucose (RsBio) and lactate assay kits (Jiancheng Bio), respectively. The glucose consumption in each experimental group was calculated as follows: Glucose consumption = glucose concentration (fresh complete medium)–glucose concentration (experimental group).

### 2.7 Determination of ATP concentration

Cells were seeded in 6‐well plates at a density of 5 × 10^5^ cells/well. Following overnight incubation at 37°C, the medium was replaced with fresh culture medium. After 6 or 24 h, in accordance with the manufacturer's instructions, cells were washed with PBS, and then, their ATP levels were determined using an ATP Assay Kit (Beyotime Institute of Biotechnology).

### 2.8 Oxygen consumption rate

Cellular oxygen consumption rate (OCR) was measured by the MitoXpress^®^ Xtra‐Oxygen Consumption Assay (Luxcel Biosciences Cork) using a CLARIOstar microplate reader (BMG Labtech). In brief, cells were plated at a density of 8 × 10^4^ cells/well in 96‐well plates (clear‐bottomed, black‐body plates) and allowed to adhere overnight at 37°C using a plate block heater. The culture medium was removed from all wells and replaced with 160 μl of pre‐warmed reaction mixture, including 10 μl of reconstituted MitoXpress^®^ Xtra reagent, 150 μl of fresh culture media with TMZ (at a final concentration of 400/800 μM) in each well. The wells were sealed by adding two drops of pre‐warmed HS Mineral Oil, and the plate was immediately analysed in a microplate reader.

### 2.9 Cell viability assays

Cells were seeded in 96‐well plates with 100µl of complete DMEM medium at a density of 1 × 10^4^ cells per well. After exposure to TMZ and/or Mdivi1 or WY14643, for 24 h, the cells in each well were incubated with MTT solution (0.5 mg/ml) dissolved in PBS for 4 h at 37°C. Then, 150 µl of DMSO was added to each well. The absorbance was measured at 570 nm using a CLARIOstar microplate reader (BMG Labtech). Cell viability was then calculated as follows: cell viability = absorbance of experimental group/absorbance of control group × 100%.

### 2.10 qPCR assay

RNA was extracted using a RNeasy kit (Qiagen) in accordance with the manufacturer's protocol. Quantitative real‐time reverse transcription‐PCR (qRT‐PCR) was then performed using a two‐step method. Data analysis was based on the delt‐delt‐Ct method using β‐Actin as a normalization control. PCR was conducted in a QuantStudio5 real‐time PCR system (Thermo Fisher) and analysed using QuantStudio Design & Analysis software v1.3.1 (Thermo Fisher).

In order to evaluate the mtDNA copy number, we extracted DNA from cells in each group and determined the copy number of the mtDNA gene ND1, as normalized to the 18S gene. In order to evaluate the extent of DNA damage, we selected two fragments of mtDNA (79bp and 230bp in length) as target templates for a qPCR array using the 18S gene as an internal reference. The extent of mtDNA damage was parallel to the value of 79bp/230bp. The primers used for the real‐time PCR are listed in Table 1.

**TABLE 1:** Primers used

Genes	Primers
TP53	Forward‐CAGCACATGACGGAGGTTGT
	Reverse‐TCATCCAAATACTCCACACGC
ND1	Forward‐CACCCAAGAACAGGGTTTGT
	Reverse‐TGGCCATGGGATTGTTGTTAA
79bp	Forward‐ CAGCCGCTATTAAAGGTTCG
	Reverse‐ CCTGGATTACTCCGGTCTGA
230bp	Forward‐ CAGCCGCTATTAAAGGTTCG
	Reverse‐ GGGCTCTGCCATCTTAACAA
18S	Forward‐TAGAGGGACAAGTGGCGTTC
	Reverse‐CGCTGAGCCAGTCAGTGT
PINK1	Forward‐GGAGGAGTATCTGATAGGGCAG
	Reverse‐AACCCGGTGCTCTTTGTCAC
β‐Actin	Forward‐CTCCATCCTGGCCTCGCTGT
	Reverse‐GCTGTCACCTTCACCGTTCC

### 2.11 The extraction of nuclear and mitochondrial proteins

The Mitochondria and Nuclear Extraction Kit for Cells (Beyotime Biotechnology) was used to isolate nuclear and mitochondrial fractions from each experimental sample. In each case, we followed the manufacturer's guidelines.

### 2.12 TP53 overexpression

A plasmid that overexpressed TP53, along with a negative control (NC) plasmid, were constructed by GenePharma Co., Ltd. Cells were transfected with the plasmids using the Thermo Scientific™ TurboFect™ Transfection Reagent in accordance with the manufacturer's protocol. In brief, cells were plated in 6‐well plates and transfected the next day with 4 μg of plasmid using 6 μl of transfection reagent. Cells were harvested 24 h after transfection, and whole‐cell lysates were isolated for Western blotting. The sequence of TP53 is as follows: ATGGAGGAGCCGCAGTCAGATCCTAGCGTCGAGCCCCCTCTGAGTCAGGAAACATTTTCAGACCTATGGAAACTACTTCCTGAAAACAACGTTCTGTCCCCCTTGCCGTCCCAAGCAATGGATGATTTGATGCTGTCCCCGGACGATATTGAACAATGGTTCACTGAAGACCCAGGTCCAGATGAAGCTCCCAGAATGCCAGAGGCTGCTCCCCCCGTGGCCCCTGCACCAGCAGCTCCTACACCGGCGGCCCCTGCACCAGCCCCCTCCTGGCCCCTGTCATCTTCTGTCCCTTCCCAGAAAACCTACCAGGGCAGCTACGGTTTCCGTCTGGGCTTCTTGCATTCTGGGACAGCCAAGTCTGTGACTTGCACGTACTCCCCTGCCCTCAACAAGATGTTTTGCCAACTGGCCAAGACCTGCCCTGTGCAGCTGTGGGTTGATTCCACACCCCCGCCCGGCACCCGCGTCCGCGCCATGGCCATCTACAAGCAGTCACAGCACATGACGGAGGTTGTGAGGCGCTGCCCCCACCATGAGCGCTGCTCAGATAGCGATGGTCTGGCCCCTCCTCAGCATCTTATCCGAGTGGAAGGAAATTTGCGTGTGGAGTATTTGGATGACAGAAACACTTTTCGACATAGTGTGGTGGTGCCCTATGAGCCGCCTGAGGTTGGCTCTGACTGTACCACCATCCACTACAACTACATGTGTAACAGTTCCTGCATGGGCGGCATGAACCGGAGGCCCATCCTCACCATCATCACACTGGAAGACTCCAGTGGTAATCTACTGGGACGGAACAGCTTTGAGGTGCGTGTTTGTGCCTGTCCTGGGAGAGACCGGCGCACAGAGGAAGAGAATCTCCGCAAGAAAGGGGAGCCTCACCACGAGCTGCCCCCAGGGAGCACTAAGCGAGCACTGCCCAACAACACCAGCTCCTCTCCCCAGCCAAAGAAGAAACCACTGGATGGAGAATATTTCACCCTTCAGATCCGTGGGCGTGAGCGCTTCGAGATGTTCCGAGAGCTGAATGAGGCCTTGGAACTCAAGGATGCCCAGGCTGGGAAGGAGCCAGGGGGGAGCAGGGCTCACTCCAGCCACCTGAAGTCCAAAAAGGGTCAGTCTACCTCCCGCCATAAAAAACTCATGTTCAAGACAGAAGGGCCTGACTCAGACTGA.

### 2.13 Immunohistochemistry

Tumour tissues were fixed in 4% paraformaldehyde for 24 h and embedded in paraffin. Paraffin sections were cut into sections (4 μm thick) and then deparaffinized and rehydrated prior to antigen retrieval. Sections were blocked with 10% bovine serum albumin in TBS‐Tween 20 (Sigma‐Aldrich) for 1 h at room temperature. Sections were then incubated overnight at 4℃ with primary antibodies against P53 (21891–1‐AP) and PINK1 (23274–1‐AP). The following morning, sections were incubated with secondary antibodies for 30 min at 37℃. Then, slides were incubated with diaminobenzidine for 5 min, and then counterstained with Gill's haematoxylin for 30 s. Images were captured on a microscope (Discover ECHO Company).

### 2.14 TEM observation

Tumour tissues from the different treatment groups were fixed in 2.5% glutaraldehyde and then embedded in an embedding agent. Next, specimens were sliced into sections (50–70 nm in thickness). The sections were then dyed with uranyl acetate and citrate dye solution. The TEM system (Thermo Fisher Scientific Company) operated at an acceleration voltage of 80 kV and an electron tomography voltage of 120 kV. Specimens were magnified by ×150,000, and images were acquired. Three tumour specimens in each treatment group were randomly selected for TEM examination. And each specimen outputted a clear picture of which the mean length of mitochondria was measured directly by the software of TEM. Then, the data were analysed.

### 2.15 Flow cytometry

Apoptosis assays were performed using an Apoptosis Detection Kit (BD Biosciences). Cells from different treatment groups were trypsinized with 0.25% trypsin, and assays were conducted in accordance with the manufacturer's protocol. In total, 10000 cells were counted in each treatment group using a Guava^®^ easyCyte flow cytometer (Merck KGaA).

For the analysis of mitochondrial mass, cells were grown in 6‐well plates and treated with the different agents. Cells were trypsinized with 0.25% trypsin and suspended cells were stained with MitoTracker™ Red FM (Invitrogen) (1:10000) for 30 min at 37℃. Cells were then rinsed three times with PBS, and 10,000 cells were counted for each treatment group using a Guava^®^ easyCyte flow cytometer (Merck KGaA).

The production of ROS was measured by a ROS Assay Kit (Beyotime Biotechnology). In brief, cells were collected and resuspended in DMEM medium after treatment and then incubated with DCFH‐DA for 30 min at 37°C. Cells were then rinsed three times in PBS, and 10,000 cells were for each treatment group using a Guava^®^ easyCyte flow cytometer (Merck KGaA).

### 2.16 The mito stress test

U87 cells were seeded at a density of 8000 cells/well and treated for 24 h with the respective treatments (Control, TMZ, Compound C, TMZ+Compound C). On the day of the assay, cell culture medium was removed and replaced by assay medium supplemented with agents according to the protocol and the Seahorse XFe96 analyzer was used. In the test, basal cellular oxygen consumption was determined, followed by injection of the ATP synthase inhibitor oligomycin (5 µM), the uncoupler fluoro‐carbonyl cyanide phenylhydrazone (FCCP) (2 µM) and the complex I inhibitor rotenone (0.5 µM) combined with the complex III inhibitor antimycin A (0.5 µM). The oxygen consumption rate was detected, and each measurement was normalized to cell content.

### 2.17 Statistical analysis

Data from three independent experiments were collected and analysed using SPSS 22.0 (IBM Corp.) and the Student's *t* test. Analysis of variance (ANOVA) was also used to conduct multiple comparisons using GraphPad Prism 7.00 (GraphPad Software, Inc.). Dunnett's post hoc multiple comparisons test was used after ANOVA. Data are presented as means ± standard deviation, and *p *< 0.05 was considered to indicate a statistically significant difference.

## 3 RESULTS

### 3.1 The level of oxidative phosphorylation and mitochondrial mass in GBM cells increased under TMZ treatment

**FIGURE 1:**
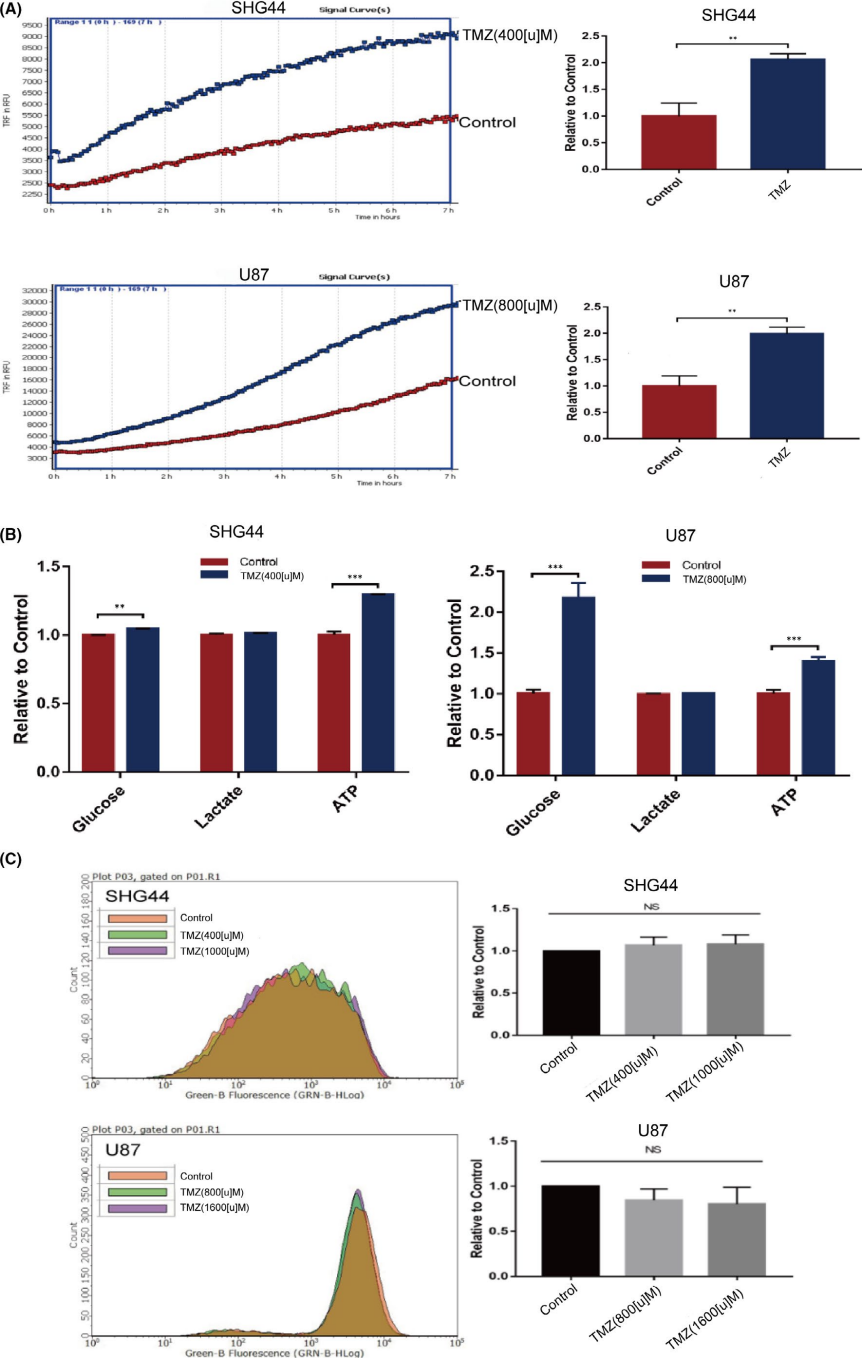
Changes of metabolic mode by TMZ treatment in GBM cells. (A) TMZ treatment induced an increase in the oxygen consumption rate of SHG44 and U87 cells; (B) TMZ treatment induced an increased glucose consumption and intracellular ATP levels in SHG44 and U87 cells within 6 h; there was no significant difference in terms of lactate secretion; (C) Under TMZ treatment, ROS levels remained steady over a period of 6 h in both SHG44 and U87 cells. (*p < 0.05, **p < 0.01, ***p < 0.001)

As an alkylating agent, TMZ can induce damage to nuclear DNA and mitochondrial DNA.^14^ It is generally believed that DNA damage, especially mitochondrial DNA damage, will affect the expression of subunits within the respiratory chain complex,^15^ thus influencing the metabolic activity of tumour cells. Using SHG44 and U87 cell lines, we first evaluated the metabolic activity of cells under TMZ treatment. When treated with TMZ, both cell lines showed increased oxygen consumption rate (Figure 1A), increased levels of intracellular ATP and glucose consumption (Figure 1B), while the lactic acid secretion (Figure 1B) and the levels of intracellular ROS remained unchanged (Figure 1C). These findings suggested that TMZ treatment increased the level of oxidative phosphorylation in SHG44 and U87 cells but without increasing the levels of ROS.

**FIGURE 2:**
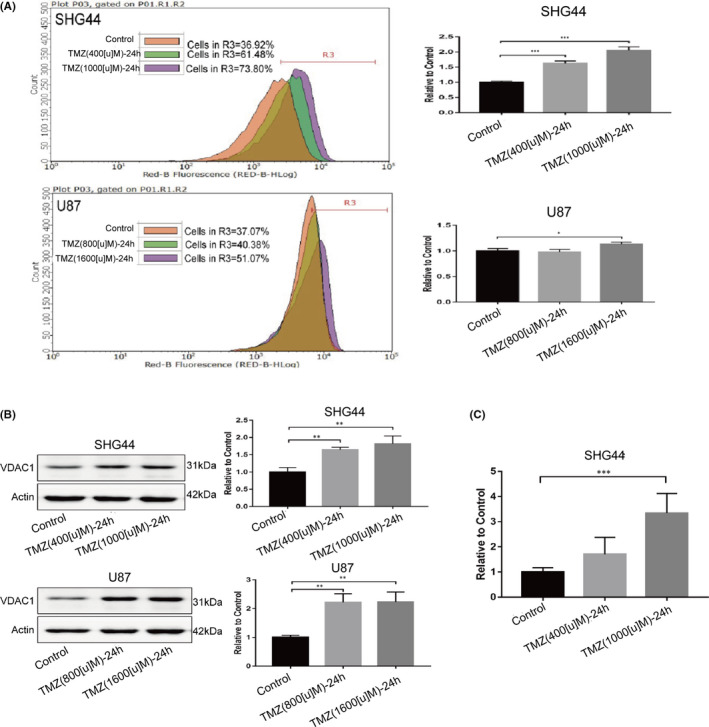
TMZ treatment increased the mitochondrial mass in SHG44 and U87 cells. (A) Mitochondrial mass was evaluated by labelling mitochondria with a Mitotracker (red) fluorescent probe. The examination of flow cytometry showed that cells in the TMZ treatment group were with the higher fluorescence intensity; (B) the WB analysis revealed that the expression levels of VDAC1 increased in the TMZ treatment group; (C) TMZ induced an increase of mitochondrial DNA copy number in SHG44 cells. (*p < 0.05, **p < 0.01, ***p < 0.001)

**FIGURE 3:**
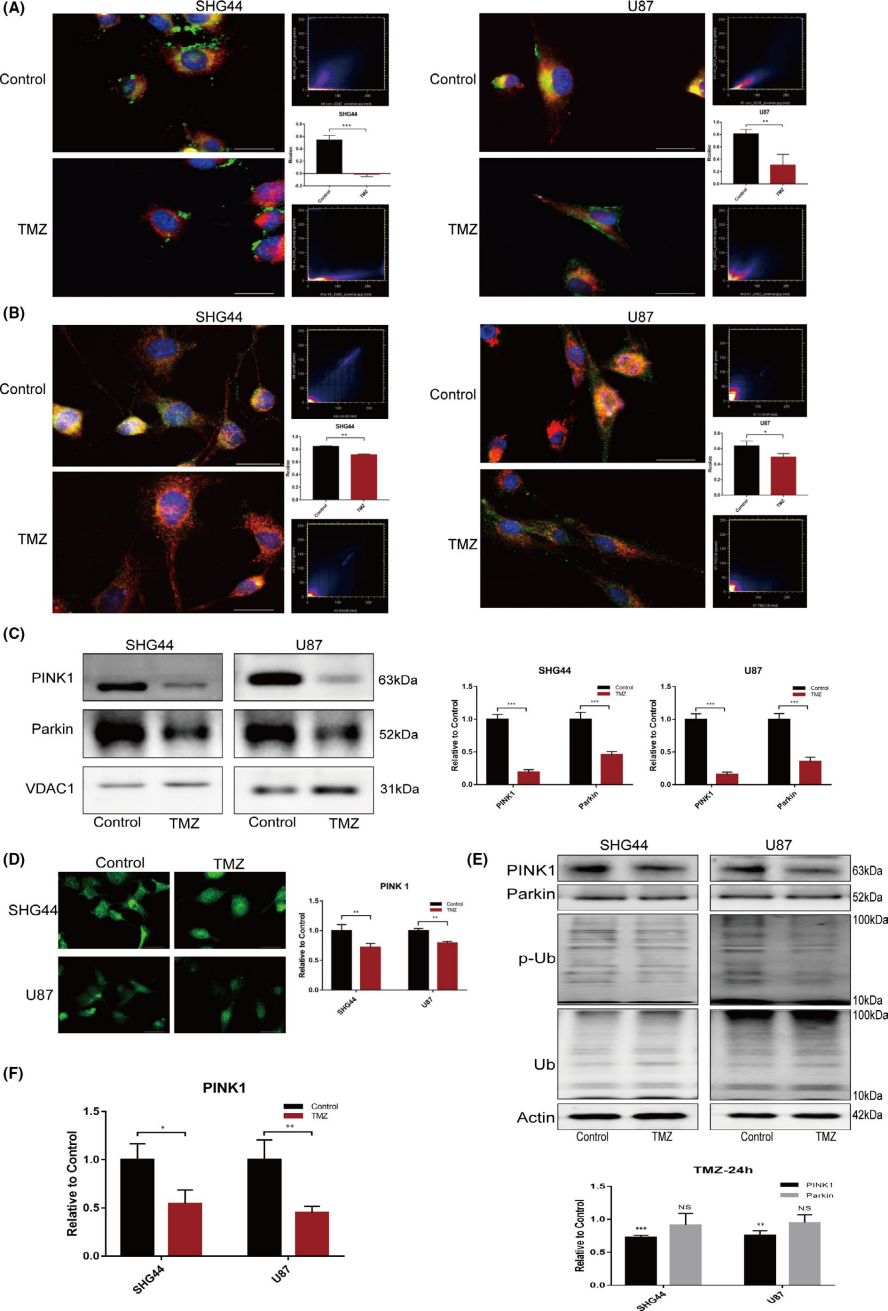
Mitophagy flux in GBM cells decreased under TMZ treatment. (A) Mitotracker (red) and Lysotracker (green) were used to label mitochondria and lysosomes, respectively. This analysis revealed a reduction in the co‐localization of mitochondria and lysosomes in the two cell lines when treated with TMZ; (B) IF experiments further showed that the co‐localization of Parkin (green) and mitochondria (red) in the TMZ group was reduced when compared to the Control; (C) Analysis of mitochondrial protein suggested that the accumulation of PINK1 and Parkin was decreased in the mitochondria of cells treated with TMZ. (D) IF assays also showed that the expression of PINK1 (green) was lower in cells that were treated with TMZ; (E) WB assays further confirmed that TMZ treatment downregulated the expression of PINK1 in both SHG44 and U87 cell lines. The specific phosphorylated product of PINK1 (phosphorylated (Ser65) ubiquitin) was also downregulated. The expression of Parkin in these cells did not change significantly. (F) The results of qPCR inferred that the PINK1 was transcriptionally downregulated under TMZ treatment. (*p < 0.05, **p < 0.01, ***p < 0.001)

In order to identify the mechanisms underlying the increased level of oxidative phosphorylation, we first detected the mass of mitochondria in the two cell lines, using three methods: (1) labelling mitochondria with specific fluorescent probes (Figure 2A), (2) by detecting the expression levels of the mitochondrial marker protein VDAC1 (Figure 2B) and (3) by detecting the copy number of mitochondrial DNA (Figure 2C). When treated with TMZ, the mass of mitochondria in SHG44 and U87 cells was significantly higher than Control group. An increase in the copy number of mitochondrial DNA was observed in SHG44 cells. Mitochondria are the only organelle in which oxidative phosphorylation can occur.^16^ Therefore, an increase in the mass of the mitochondria in response to TMZ treatment is an important factor underlying the increased level of oxidative phosphorylation.

### 3.2 Reduced mitophagic flux was an important factor underlying the increased mitochondrial mass

The mass of mitochondria within a cell is precisely controlled by mitophagy.^17^ In order to investigate whether mitochondrial dynamics is involved in metabolic reprogramming under chemotherapy stress, we evaluated the flux in mitophagy. There are many pathways that mediate mitophagy; the most dominant pathway is the PINK1‐Parkin pathway.^18^, ^19^ We first evaluated the level of the fusion between mitochondria and lysosomes in the two cell lines under TMZ treatment and found that the co‐localization of the two organelles was reduced (Figure 3A). We also studied the co‐localization of Parkin and mitochondria with IF assay (Figure 3B). And the further WB assay with mitochondrial protein revealed that when cells were treated with TMZ, the accumulation of Parkin and PINK1 in mitochondria decreased (Figure 3C). In the IF assay and WB assay with whole‐cell proteins, the expression levels of Parkin were unchanged while the expression of PINK1 and PINK1‐specific phosphorylation product (ser65) phosphorylated ubiquitin decreased when treated with TMZ (Figure 3D, E). In order to further confirm the change of (ser65) phosphorylated ubiquitin, we introduced the agent MG132 and combined it with TMZ in the treatment of SHG44 and U87 cells (Figure S1A). We also evaluated the expression of PINK1 in transcriptional level, in which, the downregulation of mRNA was observed (Figure 3F). These results suggested that the downregulation of mitophagy flux is an important factor underlying the increased mitochondrial mass. Furthermore, the downregulation of mitophagy flux may be caused by a downregulation in the expression of PINK1 transcriptionally.

### 3.3 The DNA damage and resultant TP53 overexpression played an important role in the downregulation of mitophagy flux

**FIGURE 4:**
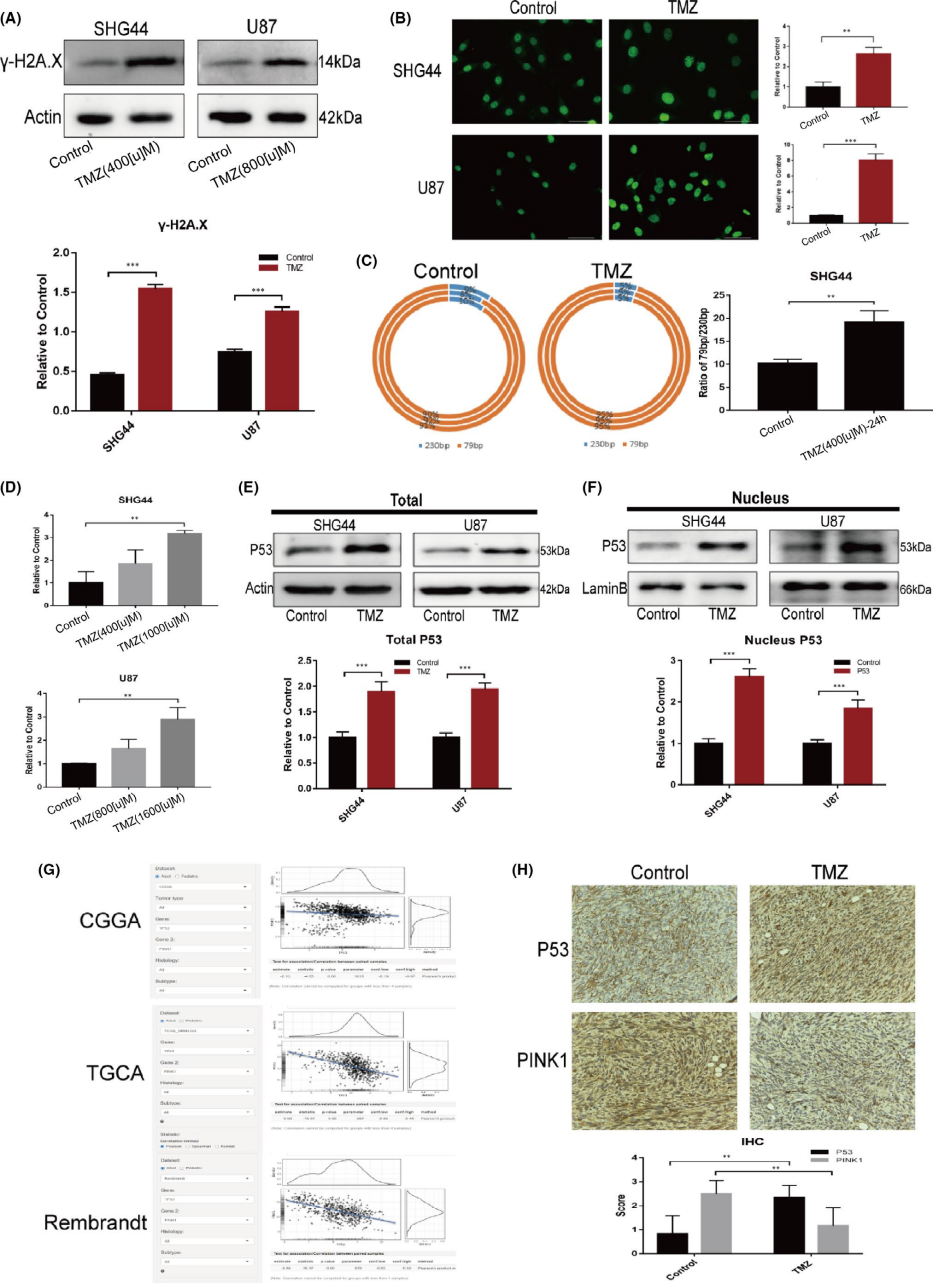
TMZ treatment‐induced DNA damage in GBM cells and the resultant TP53 overexpression affected the expression of PINK1. (A) WB assays showed that TMZ treatment increased the expression of γ‐H2A.X, a marker of cellular DNA damage. (B) IF assays also confirmed that TMZ increased the expression of γ‐H2A.X (green) in the nucleus; (C) qPCR was used to evaluate the copy number of two fragments of mitochondrial DNA (230bp fragment and 79bp fragment) and showed that in SHG44 cells, the ratio of the 79bp/230bp fragments in the TMZ treatment group was higher than that in the control group; (D) TMZ treatment led to the upregulated transcription of TP53; (E) The expression of P53 protein was also upregulated in the TMZ treatment group; (F) The combination of cell nucleus extraction and WB assays revealed an increased accumulation of P53 in the nucleus of cells undergoing TMZ treatment; (G) Analysis of the correlation between TP53 and PINK1 expression in the tumour database CGGA (R = −0.13, *p *< 0.001), TGCA (R = −0.50, *p *< 0.001) and Rembrandt (R = −0.56, *p *< 0.001) indicated that the expression levels of TP53 and PINK1 were negatively correlated in glioma; (H) In U87 cell xenograft tumour specimens, the expression levels of P53 protein and PINK1 were negatively correlated. (*p < 0.05, **p < 0.01, ***p < 0.001)

In order to further explore the mechanism by which mitophagy was downregulated, we focused on how the expression of PINK1 was regulated. We also investigated the elevated expression of intracellular nuclear transcription factor (NTF) in tumour cells under TMZ treatment. First, we found that TMZ treatment‐induced cellular DNA damage responses in both SHG44 and U87 cells; these responses involved high expression of γ‐H2A.X and the increased aggregation of the protein in the nucleus (Figure 4A, B).^20^ We also found that the copy number of the 79bp/230bp fragments of mtDNA increased under TMZ treatment in SHG44 cells, thus indicating increased levels of mtDNA damage (Figure 4C).^21^ Secondly, we observed the elevated expression of the stress response protein P53 in the two cell lines (Figure 4D, E), along with the increased accumulation of P53 protein in the nucleus (Figure 4F). As an important stress response protein in the cell, P53 can act as a transcriptional regulator in the nucleus, thus regulating the expression of a variety of downstream cell cycle monitoring genes and apoptosis‐related genes.^22^ In order to determine whether there is a correlation between the expression of P53 and PINK1, we performed correlation analysis of the expression levels of these two molecules using tumour databases CGGA, TGCA and Rembrandt. We found that the expression of these two proteins was negatively correlated (Figure 4G). These results were consistent to our immunohistochemistry analysis of tumour tissues (Figure 4H).

**FIGURE 5:**
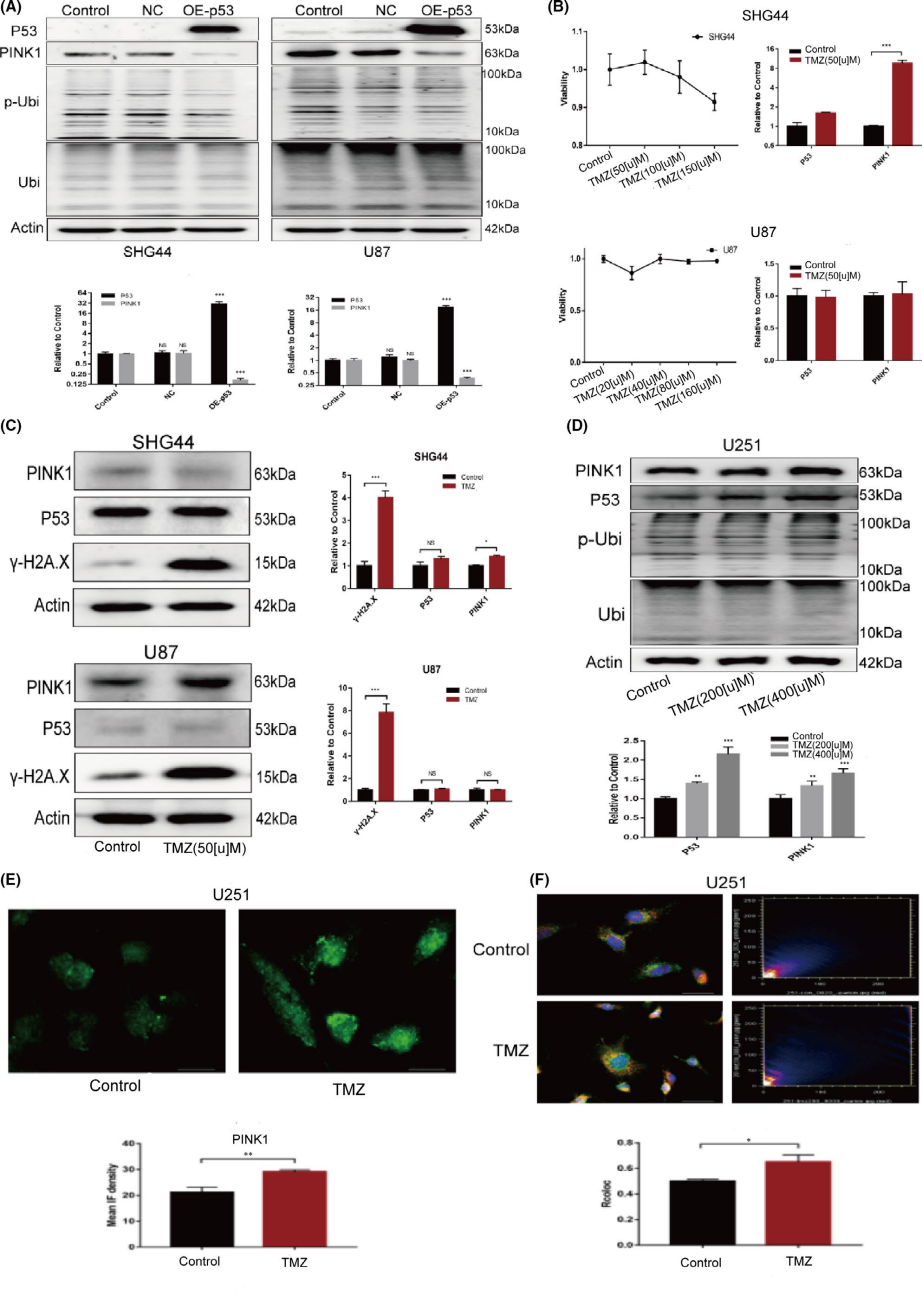
TP53 downregulated the expression of PINK1. (A) The overexpression of TP53 in SHG44 and U87 cells downregulated the expression of PINK1 and the specific phosphorylated product of PINK1 (phosphorylated(Ser65) ubiquitin); (B) SHG44 and U87 cells were treated with a low dose of TMZ (50 μM) to induce DNA damage without increasing the expression of TP53. It was found that the expression levels of PINK1 were upregulated at the transcriptional level in SHG44 cells; there was no significant change at the transcriptional level in U87 cells; (C) At the protein expression level, 50 μM of TMZ (for 24 h) induced an increased expression level of γ‐H2A.X in both cell lines, although there was no significant difference in terms of the expression of P53 and PINK1. (D) The treatment of U251 cells with different concentrations of TMZ showed that P53 expression was upregulated as the concentration of TMZ increased. The expression levels of PINK1 also increased in a concentration‐dependent manner; (E) IF assays revealed that TMZ treatment‐induced the upregulation of PINK1 expression in U251 cells; (F) Co‐localization analysis of Parkin (green) and mitochondria (red) in U251 cells showed that TMZ treatment promoted Parkin to translocate to the mitochondria. (*p < 0.05, **p<0.01, ***p < 0.001)

To further verify the regulatory effect of P53 on the expression of PINK1, we established three cell models: (1) establishing a cell model overexpressing TP53 by plasmid transfection; under this condition, we observed that PINK1 expression was downregulated in cells overexpressing TP53, and that the expression of Ser65 phosphorylated ubiquitin was correspondingly downregulated (Figure 5A); (2) treating SHG44 and U87 cells with a small dose of TMZ to induce DNA damage but without activating the expression of TP53; under this condition, we did not observe the downregulation of PINK1 (Figure 5B, C); and (3) treating the TP53‐mutated cell line U251 with TMZ.^23^ In U251 cells, the P53 protein exhibited point mutations in its DNA binding domain; in this condition, P53 protein cannot bind to nuclear DNA to act as a nuclear transcriptional regulator.^24^ In this model, we observed that TMZ treatment increased the expression of mut‐P53 protein in U251 cells, and also increased the expression of PINK1 protein in U251 cells (Figure 5D, E). What is more, the localization of Parkin to mitochondria reduced (Figure 3F). These findings confirmed that wt‐TP53 participates in the downregulation of mitophagy flux by reducing the expression levels of PINK1; this mechanism is consistent with another excellent study.^25^

### 3.4 The subcellular distribution of Drp1 impacted the mitochondrial morphology which may play role in metabolic reprogramming

**FIGURE 6:**
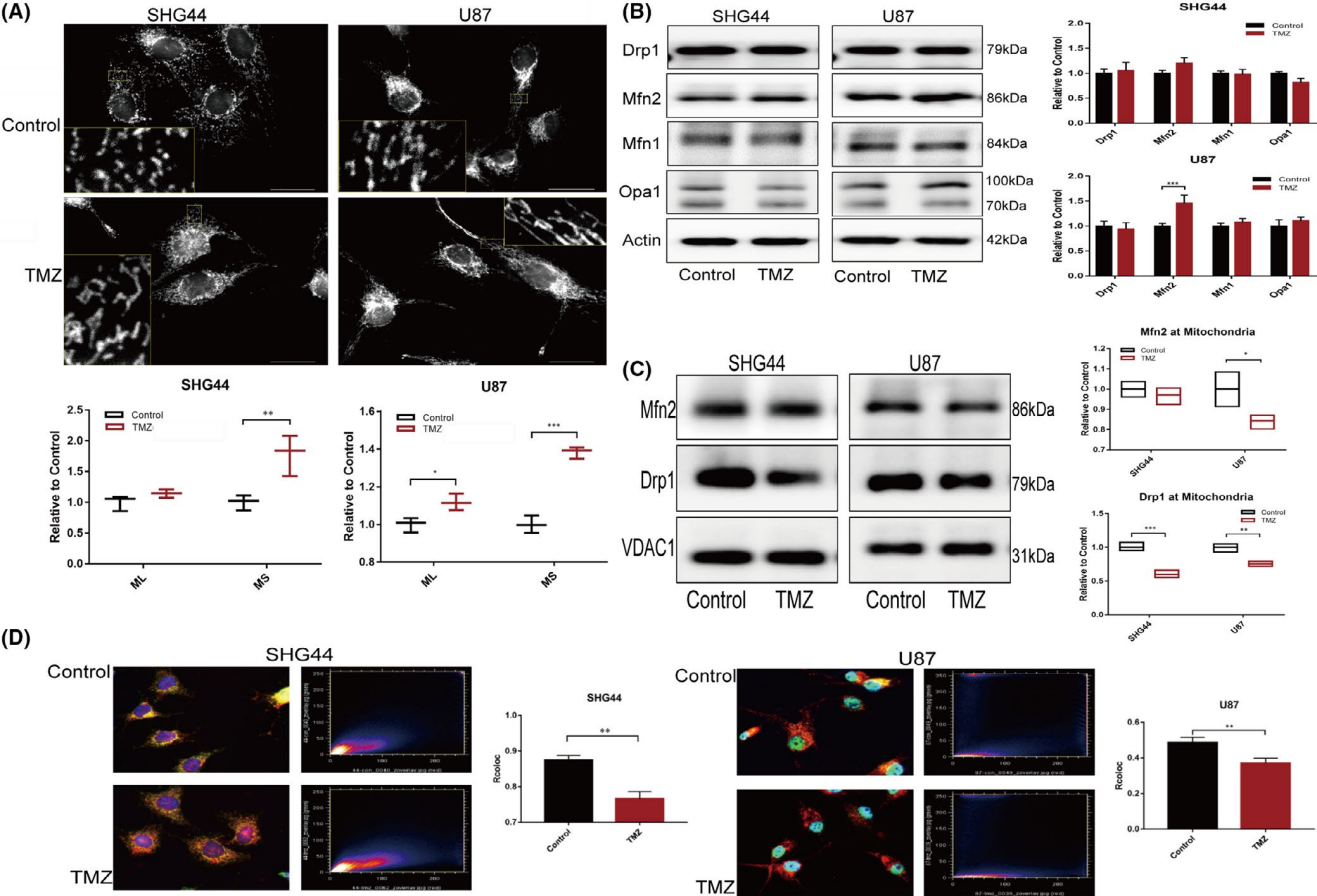
Subcellular distribution of Drp1 impacted the mitochondrial morphology. (A) It was found that the mean size of the mitochondria in SHG44 cells in the TMZ treatment group increased while the mean mitochondrial length and mean mitochondrial size in the U87 cells increased; (B) We also evaluated the expression of key proteins in the mitochondrial fission and fusion process during TMZ treatment and found that the expression levels of Drp1, Mfn1, Mfn2 and Opa1, in SHG44 cells did not change significantly; the only protein showing increased expression levels in U87 cells was Mfn2; (C) Mitochondrial protein extracts and WB assays indicated that there was no significant difference in the expression of Mfn2 in the mitochondria of SHG44 cells. The expression of Mfn2 in the mitochondria of U87 cells was downregulated, and the mitochondrial expression of Drp1 was downregulated in both SHG44 and U87 cells; (D) IF assays also confirmed that the co‐localization of Drp1 and mitochondria decreased in both cell lines following TMZ treatment. (*p < 0.05, **p < 0.01, ***p < 0.001)

It is generally believed that the downregulation of mitophagy flux is accompanied by the accumulation of damaged mitochondria, which is the main source of excessive ROS production in cells.^26^, ^27^ However, in the present study, the downregulation of mitophagy flux was not associated with elevated ROS levels in tumour cells, thus implying that there may be other mitochondrial quality control (MQC) mechanisms involved. Mitochondrial fission and fusion play important roles in MQC. Mitochondrial fusion can integrate damaged mitochondria structurally and functionally, thus, improving the efficiency of energy synthesis and reducing the synthesis of dangerous metabolites. Mitochondrial fission can remove severely damaged mitochondria from cells.^13^ First, we evaluated mitochondrial morphology under TMZ treatment and found that the mean size of mitochondria in SHG44 cells increased while the mean size and length of the mitochondria in U87 cells increased, thus indicating that the mitochondrial system had begun to undergo fusion (Figure 6 A). Mitochondrial morphology is also known to be affected by a variety of proteins involved in mitochondrial fission and fusion.^28^ We evaluated the key proteins involved in mitochondrial fission and fusion and found that TMZ treatment increased the expression levels of Mfn2 in U87 cells (Figure 6B). The expression levels of key proteins in the mitochondria further suggested that the accumulation of Mfn2 in the mitochondria of U87 cells decreased. The expression of Drp1 in the mitochondria also decreased in both cell lines (Figure 6C, D). The morphology of the mitochondria is determined by the balance between mitochondrial fission and mitochondrial fusion. The reduced translocation of Drp1 to the mitochondria was an important factor that was responsible for TMZ‐induced mitochondrial fusion in GBM cells.

### 3.5 The activated AMPK regulated the translocation of Drp1 to mitochondria

**FIGURE 7:**
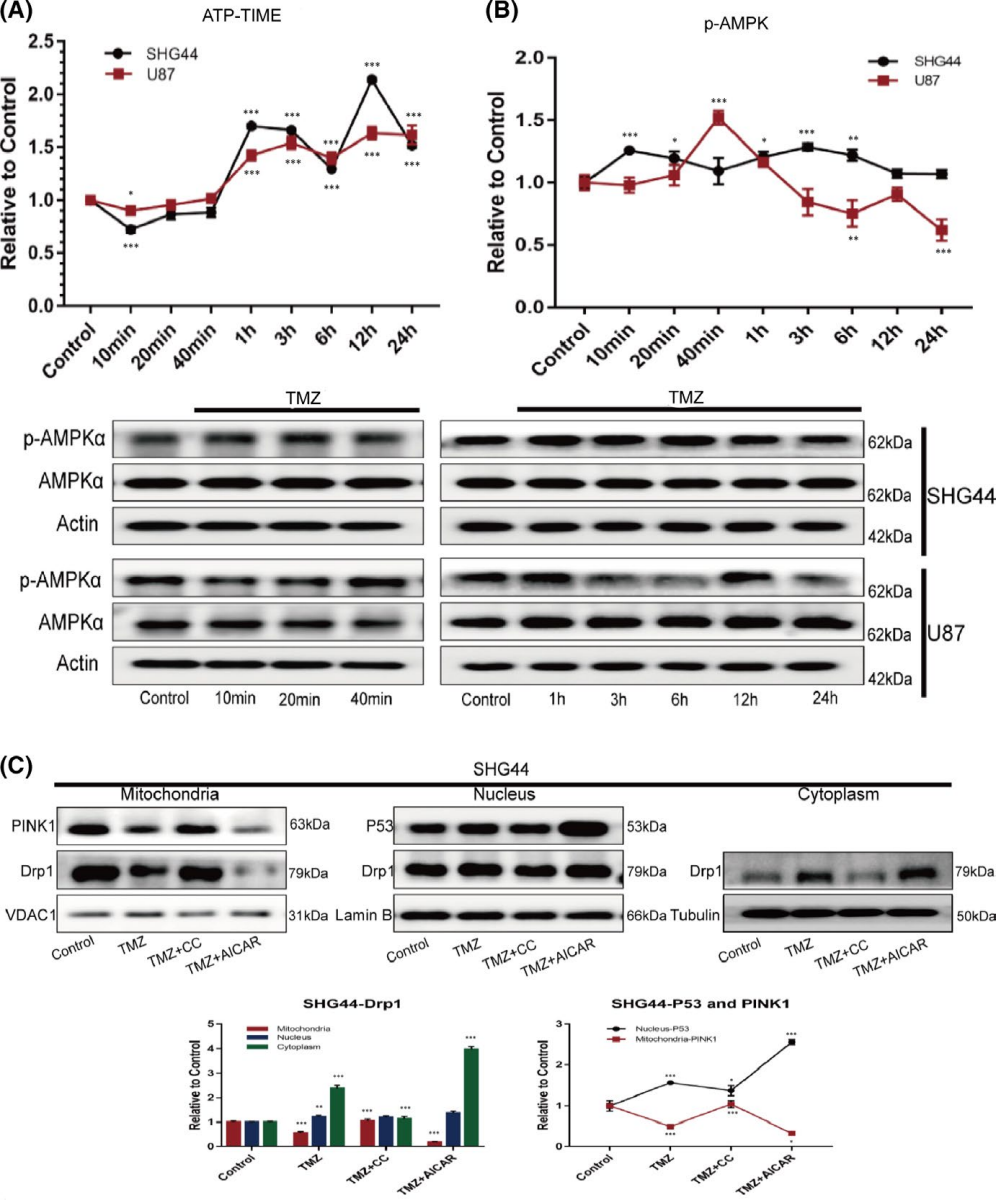
Activated AMPK regulated the translocation of Drp1 to mitochondria (A). Evaluation of ATP levels in SHG4 and U87 cells over different time courses of TMZ treatment showed that intracellular levels of ATP decreased within a short period of time after TMZ treatment; there were two troughs in the curve showing ATP concentration over a 24 h period. (B) Evaluation of the expression of phosphorylated AMPKα in tumour cells over different time courses of TMZ treatment revealed that both cell lines were upregulated within 24 h; furthermore, two peaks of phosphorylated AMPKα expression were observed during this time period. (C) In SHG44 cells, the localization of Drp1 and PINK1 in the mitochondria decreased during TMZ treatment. Drp1 and P53 increased in the nucleus, while levels of DRp1 increased in the nucleus. When treated with TMZ+CC, levels of Drp1 and PINK1 increased in the mitochondria while the levels of P53 decreased in the nucleus. Compared with the TMZ group, the levels of Drp1 in the cytoplasm decreased, levels of Drp1 and PINK1 in the mitochondria decreased, and the levels of P53 in the nucleus increased. Under the TMZ+AICAR treatment, levels of Drp1 in the cytoplasm increased significantly. (*p < 0.05, **p < 0.01, ***p < 0.001)

We were also interested in the mechanisms responsible for the reduced translocation of DRP1 to the mitochondria. We hypothesize that the purpose of metabolic reprogramming in tumour cells under chemotherapeutic stress is to satisfy the energy demand of the cells, and it must stem from the energy demand of tumour cells, so we first monitored the intracellular levels of ATP over different courses of TMZ treatment. Within a short time (10 min) after TMZ treatment, the tumour cells showed reduced levels of ATP; thereafter, however, the levels of ATP increased. The curve created by plotting ATP concentration with time showed two troughs; one at 10 min and the other at 6 h (Figure 7A). Furthermore, the expression levels of Thr172‐phosphorylated AMPKα suggested that the expression of phosphorylated AMPKα in tumour cells was upregulated in only a short period of time, and that there were two corresponding peaks in the expression‐time curve. The appearance of these peaks in U87 cells lagged behind the peaks that occurred in SHG44 cells (Figure 7B). AMPK, as an ‘energy sensor’ in cells, is an important molecule that regulates metabolism.^29^ We next considered whether AMPK participates in the metabolic reprogramming of GBM cells under TMZ chemotherapy stress by influencing mitochondrial dynamics. In order to answer this question, we used an AMPK activator, AICAR,^30^ and its function inhibitor, Compound C,^31^ as research tools. We treated GBM cells with the two agents in combination with TMZ. In SHG44 cells, TMZ treatment reduced the localization of Drp1 in the mitochondria, while more Drp1 was translocated to the cytoplasm and nucleus. When Compound C was combined with TMZ, we found that Drp1 was re‐translocated from the cytoplasm to the mitochondria; there was no significant difference in the expression levels of Drp1 in the nucleus when compared with the TMZ treatment group. There was a reduction in the concentration of Drp1 in the mitochondria after the combined use of AICAR and TMA. Drp1 was translocated to the cytoplasm, while the aggregation of Drp1 in the nucleus did not differ significantly from that in the TMZ treatment group (Figure 7C). In U87 cells, TMZ treatment promoted the translocation of Drp1 in the mitochondria and nucleus to the cytoplasm. The combination of TMZ and Compound C promoted the translocation of Drp1 from the cytoplasm and nucleus to the mitochondria. In contrast, the combination of TMZ and AICAR promoted the translocation of Drp1 from the cytoplasm to the nucleus; the expression of Drp1 in the mitochondria did not change significantly (Figure S1B). In order to explore the mechanism behind, we further evaluated the expression of the phospho(637)‐Drp1 and phospho(616)‐Drp1 and found that affecting the ratio of phospho(637)‐Drp1/phospho(616)‐Drp1 is one of the ways AMPK regulating the translocation of Drp1 to mitochondria (Figure S1C). We also found that activation of the AMPK pathway was positively correlated with the expression of P53 in the nucleus. This may be related to the phosphorylation of P53 protein by AMPK; as this leads to a reduction in the degradation of P53.^32^ We also confirmed the negative correlation between TP53 and PINK1 expression (Figure 7 C, Figure S1B). These findings suggested that AMPK regulated the translocation of Drp1 to the mitochondria under TMZ treatment, thus reducing the aggregation of Drp1 in the mitochondria. This also affected the flux in mitophagy by upregulating the expression levels of TP53 in the nucleus.

What is more, to confirm that the AMPK regulated the function of mitochondria, we performed the mito stress test by using the seahorse analyzer and revealed that: 1.TMZ elevated the OXPHOS potential of mitochondria while repressing the activity of AMPK impaired the effect (Figure S1D).

### 3.6 Interfering with mitochondrial fission and fusion affected the metabolic changes induced by TMZ

**FIGURE 8:**
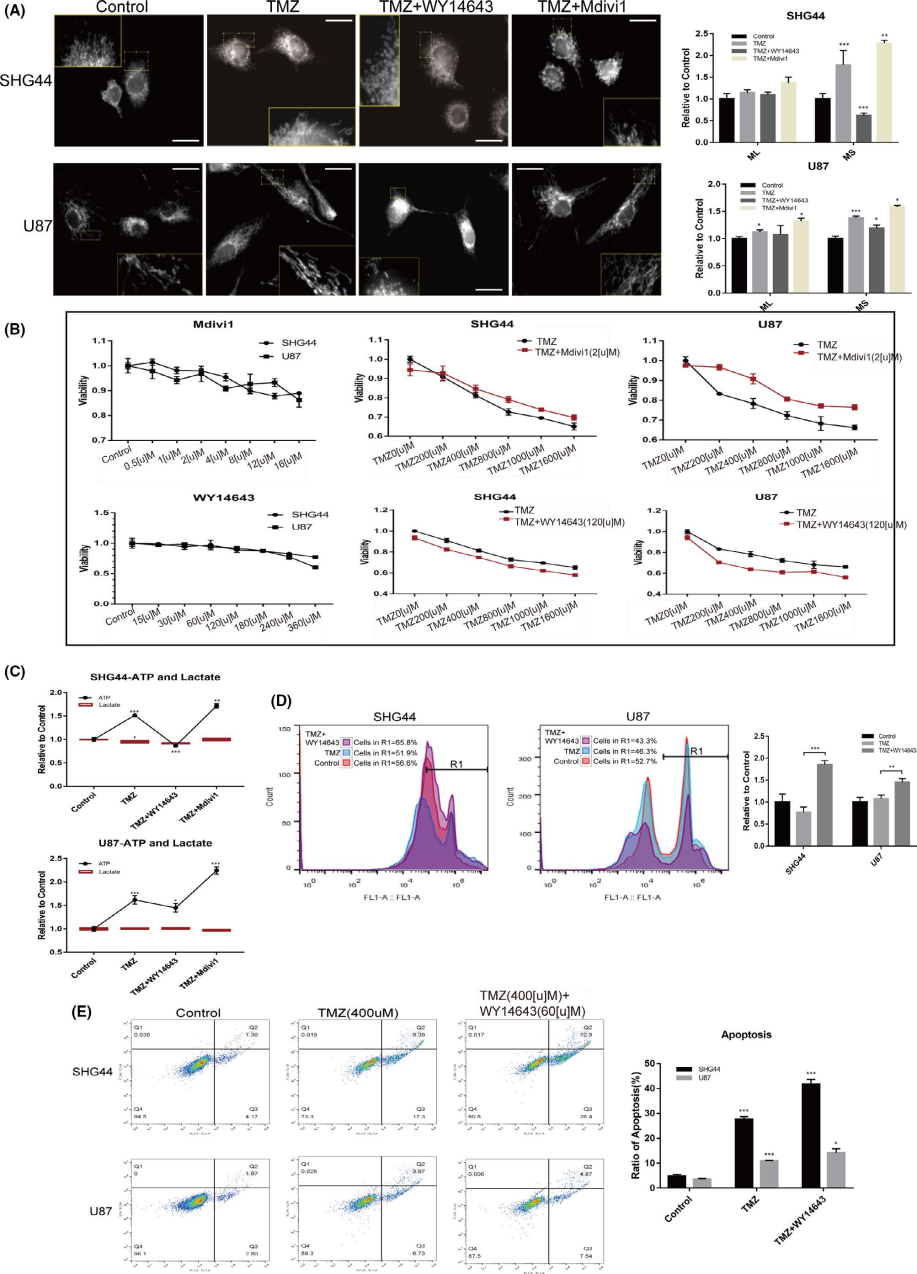
Interfering with the morphology of mitochondria influenced the metabolism of GBM and the chemoefficacy under TMZ treatment. (A) Evaluation of mitochondrial morphology under different treatments revealed that TMZ treatment increased the mean mitochondrial size, while TMZ+WY14643 treatment decreased MS compared to the TMZ group; compared to the TMZ group, TMZ+Mdivi1 treatment increased MS in SHG44 cells. For U87 cells, TMZ treatment increased mitochondrial ML and MS. TMZ+WY14643 reduced mitochondrial MS compared with the TMZ group, while TMZ+Mdivi1 increased mitochondrial ML and MS compared with the TMZ group. (B) The influence of specific concentrations of Mdivi1 and WY14643 on the TMZ concentrations‐cell viability curve of SHG44 and U87 cells. Mdivi1 drove the curve upwards while WY14643 shifted the curve downwards. (C) TMZ treatment increased the intracellular ATP levels of the two cell lines. TMZ+WY4643 reduced the levels of ATP compared with TMZ treatment, while TMZ+Mdivi1 increased the levels of ATP compared with TMZ treatment. In SHG44 cells, TMZ treatment reduced the secretion of lactate secretion. (D) There was no significant change in the ROS levels of the two cell lines after TMZ treatment for 24 h. The intracellular ROS levels of TMZ+WY14643 were significantly higher than those of the TMZ treatment group. (E) Apoptotic assays showed that the TMZ+WY14643 group exhibited a more powerful effect on apoptosis induction compared with TMZ treatment group. (*p < 0.05, **p < 0.01, ***p < 0.001)

In order to verify that the mobilization of mitochondrial dynamics was involved in the metabolic reprogramming of tumour cells under chemotherapy stress and to explore whether interfering with mitochondrial dynamics could sensitize GBM to TMZ treatment, we used two different agents: WY14643 to promote mitochondrial fission^33^, ^34^ and Mdivi1 to promote mitochondrial fusion.^35^ In the TMZ+WY14643 treatment group, we observed that the level of mitochondrial fusion was lower than that of the TMZ treatment group; the level of mitochondrial fusion in the TMZ+Mdivi1 treatment group was higher than that of the TMZ treatment group (Figure 8A). Furthermore, cell viability assays suggested that certain concentrations of WY14643 shifted the TMZ concentration‐cell viability curve of SHG44 and U87 cells downwards. Certain concentrations of Mdivi1 shifted the TMZ concentration‐cell viability curve upwards (Figure 8B). Evaluating the metabolism of different treatment groups revealed that the TMZ+WY14643 treatment group had lower intracellular levels of ATP, while the TMZ+Mdivi1 treatment group had more intracellular ATP than cells in the TMZ treatment group. However, the secretion of lactic acid did not increase significantly (Figure 8C). These results suggested that the promotion of mitochondrial fission by WY14643 partially reversed the metabolic changes induced by TMZ treatment in GBM cells. This was also supported by the detection of cellular ROS levels: the WY14643+TMZ treatment group exhibited higher levels of ROS (Figure 8D).

Finally, we found that WY14643 promoted mitochondrial fission and increased the efficacy of TMZ chemotherapy. This treatment also enhanced the efficacy of TMZ to induce apoptosis of SHG44 and U87 cells (Figure 8 E).

### 3.7 In vivo experiments confirmed that the induction of mitochondrial fission can sensitize GBM to TMZ treatment

**FIGURE 9:**
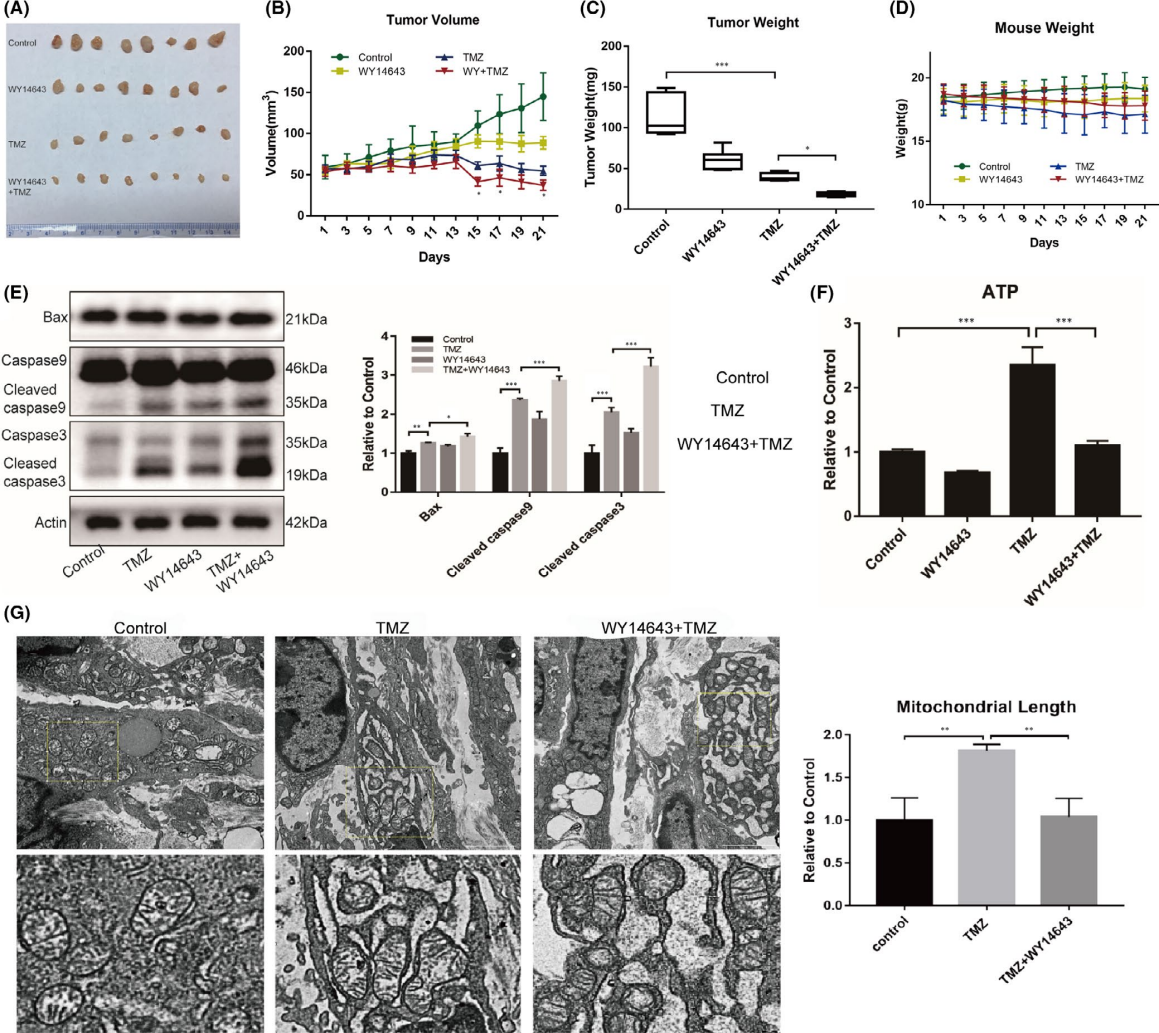
Interfering with the mitochondrial fusion enhanced the TMZ efficacy to inhibit GBM tumour growth in nude mice. (A) Images of tumours acquired from different treatment groups. The TMZ+WY14643 treatment group showed the strongest tumour suppressive effect. (B) Volume curve of xenograft tumours in different treatment groups. The TMZ+WY14643 treatment group showed the strongest levels of tumour suppression. (C) Tumour weight statistics from different treatment groups; the TMZ+WY14643 treatment group showed the strongest tumour suppressive effect; (D) Weight curve for nude mice in different treatment groups; (E) The expression of apoptosis‐related proteins in tumour tissues from different treatment groups; the expression levels of apoptosis‐related protein were the highest in the TMZ+WY14643 treatment group; (F) The evaluation of ATP levels in tumour tissues revealed that the ATP levels of the TMZ group were higher than that in the control group, while also higher than that in the TMZ+WY14643 treatment group; (G) Transmission electron microscopy examination revealed that the mean length of mitochondria in the TMZ treatment group was greater than that of the control, while the mean length of mitochondria in the TMZ+WY14643 group was less than that in the TMZ group. (*p < 0.05, **p < 0.01, ***p < 0.001)

In order to further verify that the disruption of TMZ‐induced mitochondrial fusion could sensitize GBM to TMZ chemotherapy, we designed and established a nude mouse subcutaneous xenograft tumour model, and administered WY14643 and/or TMZ. We found that the combination of WY14643 and TMZ demonstrated the most powerful inhibitory effect over tumour growth across all four groups (Figure 9A, B, C, D). Evaluation of the expression levels of apoptosis‐related proteins within tumour tissue confirmed that the highest expression levels of apoptotic proteins occurred in the group featuring the combination of two agents (Figure 9E). The evaluation of ATP levels in tumour tissues within each treatment group revealed that the ATP levels in tissues from the TMZ treatment group were higher than that in the Control group; the levels of ATP in the WY14643+TMZ group were lower than those in the TMZ treatment group (Figure 9F). The morphology of mitochondria in tumour tissue was evaluated by transmission electron microscopy (TEM) and revealed that mitochondria in the TMZ+WY14643 group had a shorter mean length than those in the TMZ treatment group (Figure 9G). These results indicated that application of WY14643 disrupted the mitochondrial fusion induced by TMZ, thus reducing ATP synthesis in tumour tissues, and increasing the efficacy of TMZ treatment.

## 4 DISCUSSION

TMZ chemotherapy is an indispensable form of treatment for GBM, and the precise efficacy of it has been confirmed in clinical studies featuring large numbers of patients.^1^ However, when used clinically, the effect of TMZ can be impaired by chemo‐resistance. Under the stress conditions created by chemotherapy, a variety of stress responses can be induced in tumour cells, including the DNA damage response (DDR), the unfolded protein response (UPR) and the upregulation of autophagy. These responses promote the survival of tumour cells.^36^ And it has been found that when tumour cells become resistant to certain chemotherapeutic agents, they usually show resistance to a variety of chemotherapeutics. This phenomenon is known as multi‐drug resistance (MDR).^37^ The exact mechanism underlying MDR has yet to be clarified; however, it reminds us that there may exist some more fundamental mechanisms in the chemo‐resistance. These mechanisms are important components of the mechanism responsible for resistance to chemotherapy in tumour cells. Energy metabolism is the basis of all cellular activities and provides support for a variety of responses to stress in cells. Deciphering the mechanisms responsible for energetic changes in the metabolism of a tumour undergoing chemotherapy stress will provide us with new concepts for elucidating MDR.

TMZ can induce DNA damage in the nucleus and the mitochondria.^38^ This DNA damage is extensive and non‐specific and is clearly likely to have a significant effect on mitochondrial function, thereby reprogramming metabolism. In this study, we first evaluated the metabolic pattern of GBM cells under TMZ treatment and found that the TMZ promoted oxidative phosphorylation. Oxidative phosphorylation is the main route by which energy is synthesized in eukaryotic cells. Although the Warburg effect has been reported in some types of tumour cell lines, aerobic respiration is still an important way of acquiring energy in tumour cells.^39^ However, increased levels of oxidative phosphorylation may also have a negative impact on the physiological activities of cells. Inefficient oxidative phosphorylation is often accompanied by the leakage of electrons and the accumulation of intracellular ROS. ROS is known to damage DNA, promote MPT opening and induce cell death.^40^ However, the increased level of oxidative phosphorylation observed in the present study was not accompanied by an increase in ROS, thus suggesting that there may be a complex control mechanism regulating mitochondrial quality in tumour cells under TMZ treatment. We also found that TMZ treatment‐induced DNA damage within tumour cells, thus increasing the expression levels of P53, a stress reactive protein. The wt‐P53 protein was found to play a role in the regulation of transcription in the nucleus and downregulated the expression of PINK1. As a key molecule in the mitophagy pathway, the downregulation of PINK1 expression also reduced the levels of its phosphorylation product, Ser65 phosphorylated ubiquitin. In turn, this led to a reduction in the recruitment of Parkin to the mitochondria and a reduction in mitophagy flux, thus resulting in the accumulation of mitochondria in tumour cells.^41^ Although the downregulation of mitophagy provided a material basis for the increase in oxidative phosphorylation, this also meant that a greater number of damaged mitochondria began to accumulate. Secondly, during the short period of TMZ treatment, there was an imbalance in ATP supply/demand and the activation of AMPK in tumour cells. On the one hand, activated AMPK further reduced the flux in mitophagy by increasing the expression of P53 protein in the nucleus; on the other hand, it reduced the translocation of Drp1 to the mitochondria, thereby increasing the level of mitochondrial fusion. Mitochondrial fusion promotes the complementarity between damaged mitochondrial from both a structural and functional point of view, thus optimizing the quality of the mitochondrial system, improving the efficiency of mitochondrial metabolism and reducing the synthesis of harmful metabolites.^13^ A number of studies have confirmed that mitochondrial fusion plays a ‘survival‐promoting’ role under stressful conditions.^42^, ^43^

Studies have shown that the increased levels of mitochondrial fusion in tumour cells are closely related to chemo‐resistance and that the promotion of mitochondrial fission enhances the efficacy of chemo‐agents to induce apoptosis^44^; these previous findings concurred with our present results. In order to further verify these results and explore a new form of chemotherapy for GBM, we tried to reverse the TMZ‐induced energy reprogramming process by interfering with mitochondrial dynamics. We found that Mdivi1 treatment promoted mitochondrial fusion and increased the level of ATP in tumour cells. In contrast, WY14643 was used to promote the fission of mitochondria and appeared to off‐set the upregulation of ATP levels to a certain extent and increased ROS levels in tumour cells under TMZ treatment. The use of WY14643 increased the sensitivity of GBM cells to TMZ chemotherapy; this was reflected in the observed increase in the proportion of tumour cells undergoing apoptosis in the TMZ+WY14643 treatment group. In the nude mouse xenograft tumour model of GBM, the tumours formed in mice from the WY14643+TMZ treatment group had a shorter mean mitochondrial length, a lower tissue ATP level, a higher expression level of apoptosis‐related proteins, and more significant growth inhibition, than those in the TMZ treatment group. This indicated that by disrupting mitochondrial dynamics, we may be able to overcome chemo‐resistance in GBM. However, the precise effect of mitochondrial fission on cell fate remains inconclusive. Some studies have found that inducing mitochondrial fission promotes the occurrence of apoptosis,^45^, ^46^ while other studies have found that mitochondrial fission exhibited anti‐apoptotic properties.^47^ These differences may be related to differences in cell type and experimental conditions. In support of the fact that mitochondrial fission may promote apoptosis, it is believed that Drp1 promotes the release of cytochrome c and the activation of Caspases.^48^ Studies have also suggested that Drp1 may be associated with the Bcl2 family, by promoting the pro‐apoptotic protein Bax to translocate to the mitochondria,^49^ or because the interaction of Drp1 with Bcl2 promotes cell apoptosis.^50^ In the present study, we found that under TMZ treatment, mitochondrial fission influenced the response of GBM cells to chemotherapy by affecting energy metabolism.

We should also highlight the fact that genetic polymorphisms exist in GBM cells. The frequency of TP53 mutations is known to be significantly different when compared between primary and recurrent glioblastomas,^51^ and that the frequency of TP53 mutations also differs across different subtypes of GBM, accounting for 54%, 32%, 21% and 0% of Proneural, Interstitial, Neuro and Classical GBM, respectively.^52^ However, wt‐TP53 and mut‐TP53 play different roles in the regulation of mitochondrial dynamics. This is reflected in our study of the TP53 mutant cell line, U251. The regulatory function of mut‐TP53 on mitochondrial dynamics needs to be investigated further. In addition, the specific molecular mechanisms underlying the regulation of AMPK signalling and translocation of Drp1 between subcellular structures, also needs to be further clarified.

## 5 CONCLUSION

The study of the mechanisms underlying chemo‐resistance has always been popular in the field of chemotherapy. Recent research studies have revealed a diverse and complex array of drug resistance mechanisms in tumour cells. In the present study, we identified a new mechanism of chemo‐resistance, the reprogramming of metabolism. By disrupting mitochondrial dynamics, it was possible to reduce the sensitivity of GBM cells to TMZ; these findings were confirmed by both *in vitro* and *in vivo* studies. This study provides us with a new concept for basic and clinical research into the chemotherapeutic strategies used to treat GBM.

## CONFLICT OF INTEREST

The authors confirm that there are no conflicts of interest.

## AUTHOR CONTRIBUTIONS

**Nan Wang:** Writing – original draft (lead). **Renxuan Huang:** Software (equal); Visualization (equal). **Kunmeng Yang:** Data curation (equal). **Yichun He:** Writing – review & editing (equal). **Delu Dong:** Supervision (equal). **Yufei Gao:** Conceptualization (lead); Funding acquisition (lead).

## Supporting information

Fig S1

## Data Availability

The data that support the findings of this study are available on request from the corresponding author.
