# Supplementary figures and images for: Interfering with mitochondrial dynamics sensitizes glioblastoma multiforme to temozolomide chemotherapy
Source: J Cell Mol Med. 2021 Dec 28;26(3):893–912. doi: 10.1111/jcmm.17147 (PMC8817126; doi:10.1111/jcmm.17147)

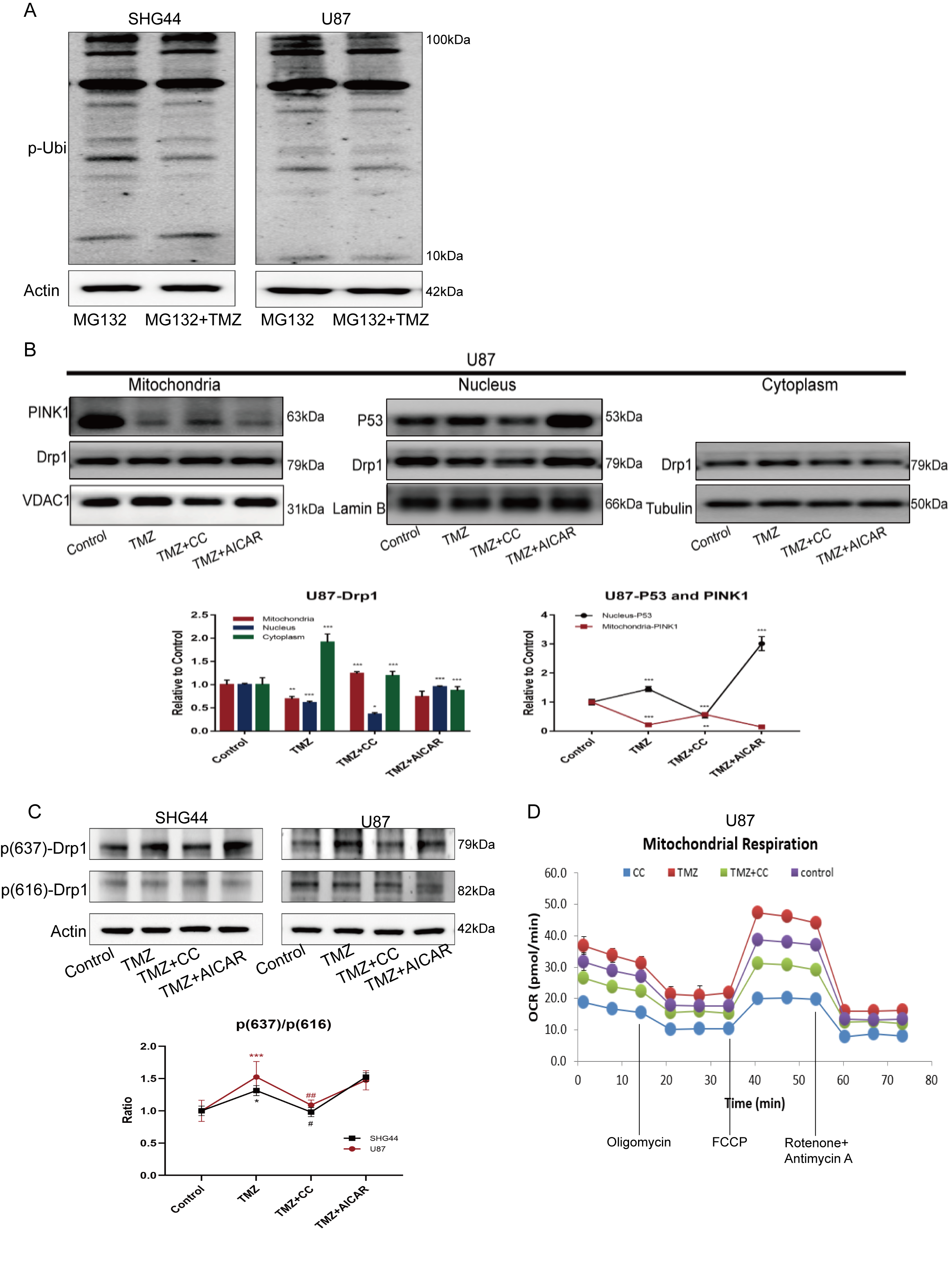

Supplement: Supplementary file 1 — Fig S1 [file JCMM-26-893-s001.png]
